# Isolation and Characterization of Multipotent Turkey Tendon-Derived Stem Cells

**DOI:** 10.1155/2018/3697971

**Published:** 2018-06-06

**Authors:** Qian Liu, Yaxi Zhu, Peter C. Amadio, Steven L. Moran, Anne Gingery, Chunfeng Zhao

**Affiliations:** ^1^Department of Orthopedic Surgery, Mayo Clinic, Rochester, MN, USA; ^2^Department of Orthopaedics, The Second Xiangya Hospital of Central South University, Changsha, China; ^3^Department of Molecular Medicine, Mayo Clinic, Rochester, MN, USA

## Abstract

Tendon injuries are among the most common and severe hand injuries with a high demand for functional recovery. Stem cells have been identified and isolated from different species and a variety of tissues for the sake of regenerative medicine. Recently, turkey has been suggested as a potential new large animal model for flexor tendon-related research. However, turkey tissue-specific stem cells have not been investigated. Here, we presented the isolation and verification of tendon-derived stem cells (TDSCs) from 6- to 8-month-old heritage-breed turkey. TDSCs were isolated from turkey flexor tendon by plating nucleated cells at the determined optimal density. Approximately 4% of the nucleated cells demonstrated clonogenicity, high proliferation rate, and trilineage differentiation potential after induction culturing. These cells expressed surface antigens CD90, CD105, and CD44, but did not express CD45. There was a high level of gene expression of tenogenic markers in TDSCs, including mohawk, collagen type I, tenascin C, and elastin. Turkey TDSCs also expressed transcription factors *PouV*, *Nanog*, and *Sox2*, which are critically involved in the regulation of stemness. The successful isolation of tendon-derived stem cells from turkey was beneficial for future studies in tendon tissue engineering and would help in the development of new treatment for tendon diseases using this novel animal model.

## 1. Introduction

Tendon injuries debilitate numerous people in athletic and occupational surroundings and remain a clinical challenge [1–3]. Injured tendon tissue heals very slowly, especially flexor tendons in the hand which is one of the most common injuries in upper extremity [4–6]. Clayton and Court-Brown studied 2794 tendinous or ligamentous injuries and found that hand tendon injuries accounted for over 1/3 of all cases [1]. Surgical interventions following flexor tendon injury are needed to restore function [7, 8] but often associated with inferior structural integrity and mechanical strength [9, 10]. Currently, stem cell-mediated approaches play a crucial role in regeneration medicine to improve the outcome of tendon injuries [11–14]. Mesenchymal stem cells (MSCs) possess clonogenicity, multipotency, and high proliferative capacity. MSCs are capable of adhering to plastic culture and can differentiate towards osteogenic, chondrogenic, and adipogenic lineages [15]. Therefore, MSCs serve as a favorable cell source for applications in the field of regenerative medicine and tissue engineering. MSCs can be isolated from several tissues such as synovium [16], umbilical cord [17], adipose [18], cartilage [19], and periosteum [20] but most commonly from bone marrow [21] and adipose tissue [22].

In addition to MSCs from bone marrow [11, 13, 14], tendon-derived stem cells (TDSCs) are emerging as a better candidate for application in tissue regenerative medicine [23–25]. TDSCs are a unique cell population present within the tendon tissues that have self-renewal and multilineage differential potential [26]. Compared with bone marrow-derived stem cells (BMSCs), TDSCs have been demonstrated to form more colonies, proliferate faster, and exhibit higher multilineage differentiation potential [26–28]. Moreover, TDSCs have been found to express tenogenic markers with increased collagen synthesis in cell culture, which makes them superior for tendon injuries repair than BMSCs [29].

Canine and chicken are currently the most popular animal models for flexor tendon-related research. However, they have several disadvantages that demand development of a new large animal model [30]. The canine model disadvantages include the cost and concerns regarding companion animal use. Although the chicken model is more affordable and has similarity to human vasculature [31, 32], it is complicated by an additional phalanx in the third digit and difficulties in postoperative rehabilitation [33]. Recently, our group has shown that turkey flexor tendons have many similarities such as anatomy and biomechanical properties to human flexor tendons, which would make the turkey a potential new large animal model for clinically relevant flexor tendon research [34]. The ability to identify turkey TDSCs would pave the way for studies on its role in tendon physiology and tendinopathy and open up new treatment for tendon diseases.

In this study, the isolation and verification of stem cells from heritage-breed turkeys' flexor tendon are evaluated. We assessed TDSCs' ability to form colonies, proliferative capacity, and morphology change. Stem cell markers were examined by electrophoresis. The multilineage potential of TDSCs was investigated by histological assay and gene expression analysis.

## 2. Materials and Methods

### 2.1. Turkey TDSC Isolation and Expansion

Three 6- to 8-month-old heritage-breed turkeys, weighing 8–10 kg, were used for the isolation of TDCSs. All animal protocols were approved by our Institutional Animal Care and Use Committee (IACUC). After euthanasia, the intact flexor digitorum profundus (FDP) tendon in the zone II area where the tendon is located within the flexor sheath was dissected out from the third digit of each turkey. The tendon sheath and peritendinous tissue were carefully removed. The tendon midsubstance was then gently cut into small pieces and digested with collagenase type I (Sigma-Aldrich, St. Louis, MO) at the concentration of 3 mg/ml for 2.5 h at 37°C and filtered through a 70 *μ*m cell strainer (BD Falcon, Bedford, MA) to remove undigested tissue. After centrifugation at 300*g* for 5 min, the cell pellet was resuspended in Dulbecco's modified Eagle's medium (DMEM; Gibco, Carlsbad, CA) containing 1% antibiotics (antibiotic–antimycotic; Gibco) and 10% fetal bovine serum (FBS). The cells were plated in 100 mm Corning® dishes at a low density (500 cells/cm^2^) and cultured at 37°C with 5% CO_2_. Nonadherent cells were removed with PBS wash after 48 h of plating. The medium was changed every 3 days. When the cultured primary cells reached 70%–80% confluence, they were subcultured after digestion with 0.25% trypsin/1 mM EDTA and used for further studies.

### 2.2. Colony-Forming Unit (CFU) Assay

For the isolation of stem cells from tendon, the optimal cell seeding density was determined by culturing nucleated cells obtained from turkey flexor tendon in 6-well plates at 50, 500, and 5000 cells/cm^2^ and the procedure was repeated in triplicate. 10 days after culture, the cells were stained with 0.5% crystal violet (Sigma, St. Louis, MO) after fixation with 4% paraformaldehyde to quantify the colony formation. Colonies larger than 2 mm in diameter and were distinguishable were included for counting. The optimal cell seeding density was determined based on the largest number of colonies obtained without contact inhibition between colonies [35]. The percentage of tendon-derived stem cells was calculated by dividing the colony number at the optimal seeding density by the nucleated cell number.

### 2.3. Cell Proliferation of Turkey TDSCs

P3 tendon-derived cells were plated in 12-well plates at 5000 cells/cm^2^ in triplicate and cultured at 37°C, 5% CO_2_. Cell proliferation was evaluated every 2 days until day 12 after cell seeding. Viable cells were determined by using Trypan blue staining. The proliferative potential of cells was presented in relative fold change.

### 2.4. RNA Isolation and Gene Expression

The gene expression of osteogenic, adipogenic, and chondrogenic markers after induction and embryonic stem cell (ESC) markers at different cell passages was examined by quantitative real-time polymerase chain reaction (qRT-PCR). The mRNA expression of tendon-related markers was also examined. Total RNA was isolated using TRIzol® reagent (Invitrogen, Grand Island, NY) per the manufacturer's protocol. RNA concentration was assessed by absorbance at 260 and 280 nm with a DS-11 spectrophotometer (DeNovix, Wilmington, DE). Complementary DNA (cDNA) was synthesized from equal amounts of RNA (1 *μ*g) using the iScript™ cDNA Synthesis Kit (Bio-Rad). All reactions were performed using SYBR Green PCR Master Mix (Applied Biosystems, Foster City, CA) on a C1000 Touch™ Thermal Cycler (Bio-Rad Laboratories, Hercules, CA) for the following genes: scleraxis (*SCX*), mohawk (*MKX*), tenomodulin (*TNMD*), thrombospondin-4 (*THBS4*), tenascin C (*TNC*), collagen type I (*COL1A1*), decorin (*DCN*), elastin (*ELN*), peroxisome proliferator-activated receptor (*PPARγ*), adipocyte-binding protein 2 (*aP2*), runt-related transcription factor 2 (*RUNX2*), osteopontin (*SPP1*), osteocalcin (*BGLAP*), sex-determining region Y-box9 (*SOX9*), collagen type II (*COL2A1*), aggrecan (*ACAN*), *PouV*, *Nanog*, and *Sox2*. The cycling program was 2 min at 95°C, then 40 cycles of amplifications, 5 s at 95°C for denaturation, 5 s at 65°C for annealing, and 5 s at 95°C for extension. PCR primers were designed using Primer3 version 0.4.0 software (Table 1). All primers were from chicken. Each sample was analyzed in triplicate. The gene expression level of the target genes was normalized to *GAPDH* and then analyzed by the 2^−∆Ct^ formula with reference to the noninduced controls. The experiment was performed in duplicates of cells from two turkeys.

### 2.5. MSC Marker Analysis

MSC surface markers, including CD90, CD105, and CD44, were examined as previously described [36]. Hematopoietic cell marker CD45 was also examined to exclude the contamination of hematopoietic cells. Briefly, a total of 10 *μ*L amplified DNA fragments were electrophoresed on a 1.5% agarose gel to detect PCR products for each marker.

### 2.6. Multidifferentiation Potential

We investigated the multipotency of P3 turkey tendon-derived cells based on the method of Pittenger et al. [37] and Scharstuhl et al. [38] with minor modifications.

#### 2.6.1. Osteogenic Differentiation

Tendon-derived cells were cultured in complete medium in a six-well plate at a density of 4 × 10^3^ cells/cm^2^. Osteogenesis was tested by inducing the cells in osteogenic differentiation medium (Gibco; StemPro® Osteogenesis Differentiation Kit) for 3 weeks. Control groups were cultured in basal complete media. The medium was refreshed every 3–4 days. To assess osteogenesis, calcium nodules were stained with Alizarin Red S after 21 days, and the osteogenic lineage-specific gene expressions (*RUNX2*, *SPP1*, and *BGLAP*) were assessed using qRT-PCR. Alizarin Red S staining was conducted by washing the cells with PBS, then fixed with 70% ethanol for 10 min, and incubated with 0.5% Alizarin Red S (pH 4.1; Sigma-Aldrich) for 30 min. Images of stained cells were obtained using a light microscope (BH2, Olympus).

#### 2.6.2. Adipogenic Differentiation

Tendon-derived cells were cultured in complete medium in a six-well plate at a density of 4 × 10^3^ cells/cm^2^. When cells reached 100% confluence, adipogenesis was induced by replacing basal medium with adipogenic differentiation medium (Gibco; StemPro Adipogenesis Differentiation Kit). After three weeks, the gene expression of adipogenic markers (*PPARγ*, *aP2*) and the accumulation of lipid droplets were assessed by qRT-PCR and Oil red-O (Sigma-Aldrich) staining, respectively. Cells cultured in basal complete medium only served as control. Fresh medium was fed to cultures every 3–4 days. Oil red-O staining was completed. Cells were washed two times with dH_2_O; filtered 0.3% Oil red-O solution was added and incubated for 15 min after fixed with 70% ethanol for 20 s. Cultures were washed with PBS three times, hematoxylin was added, and cells were incubated for 30 s. Images of stained cells were viewed using a light microscope (BH2, Olympus).

#### 2.6.3. Chondrogenic Differentiation

Micromass culture was used for inducing chondrogenesis. Briefly, 5 *μ*L droplets of cell solution were seeded in the center of 24-well plates after resuspending cells in chondrogenic differentiation medium (Gibco; StemPro Chondrogenesis Differentiation Kit) at 1.6 × 10^7^ cells/mL. After incubating for 2 hours, a 500 *μ*L chondrogenic differentiation medium (Gibco; StemPro Chondrogenesis Differentiation Kit) was added. Cultures were fed every 2–3 days. After 21 days of culture, the micromass was rinsed with PBS and fixed in 4% paraformaldehyde for 30 min. The micromass was then stained with 1% Alcian blue solution to evaluate glycosaminoglycan synthesis. The gene expressions of *COL2A1*, *SOX9*, and *ACAN* were assessed using qRT-PCR as described above.

## 3. Data Analysis

All data are presented as mean ± standard deviation. Comparison of two groups was done using two-tailed, unpaired Student's *t*-test, and the comparison of multiple groups was done using one-way factorial analysis of variance (ANOVA) followed by comparison of individual means with Tukey's test. Statistical analyses were performed with SPSS statistical software (version 17.0, SPSS Inc., Chicago, IL). *P* < 0.05 was regarded as statistically significant.

## 4. Results

### 4.1. Clonogenicity and Proliferation of Tendon-Derived Cells

**Figure 1:**
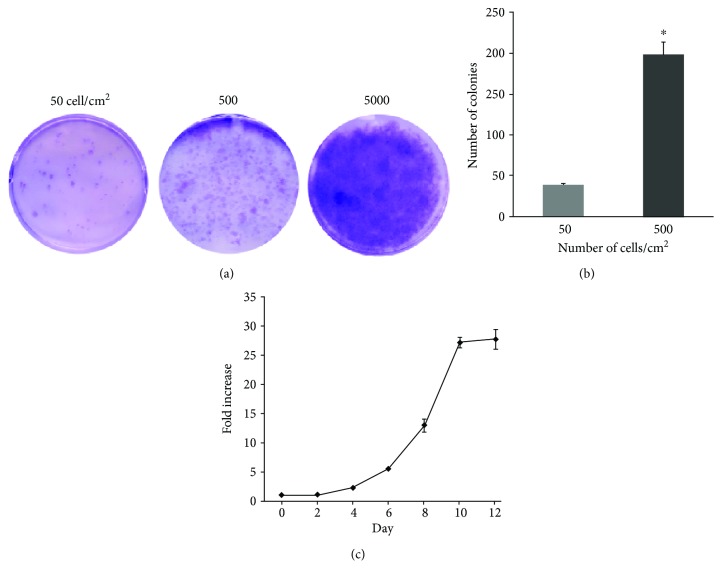
(a) Colony-forming unit assay of tendon-derived cells after 10 days of culture at 50, 500, and 5000 cells/cm^2^. (b) Number of cell colonies when tendon-derived cells were plated at 50 or 500 cells/cm^2^. *n* = 3, ^∗^*P* < 0.01. (c) Graph showing the proliferative over time of tendon-derived cells at P3. The results shown here were mean ± standard deviation of three wells for each time point. The experiment was performed independently in two turkeys.

The clonogenic capacity of tendon-derived cells was assessed using *CFU* assay. After 10 days, cells isolated from tendon formed adherent cell colonies (Figure 1(a)). The optimal cell seeding density was determined by plating cells isolated from turkey tendon at several densities. We found that at 5000 cells/cm^2^, the colonies were indistinguishable. The number of colonies was significantly higher when plating at 500 cells/cm^2^ compared to that at 50 cells/cm^2^ (198 ± 15.7 colonies versus 39 ± 1.5 colonies, *n* = 3, *P* < 0.01) (Figure 1(b)). Approximately 4% of tendon-derived nucleated cells were able to form colonies. The proliferation profile of tendon-derived cells was assessed by counting viable cells for 12 days at a 2-day interval using Trypan blue exclusion method. The cells demonstrated a more than 25-fold increase with time up to day 12, indicating that the tendon-derived cells possessed high proliferative capability (Figure 1(c)).

### 4.2. Cell Morphology of Tendon-Derived Cells

**Figure 2:**
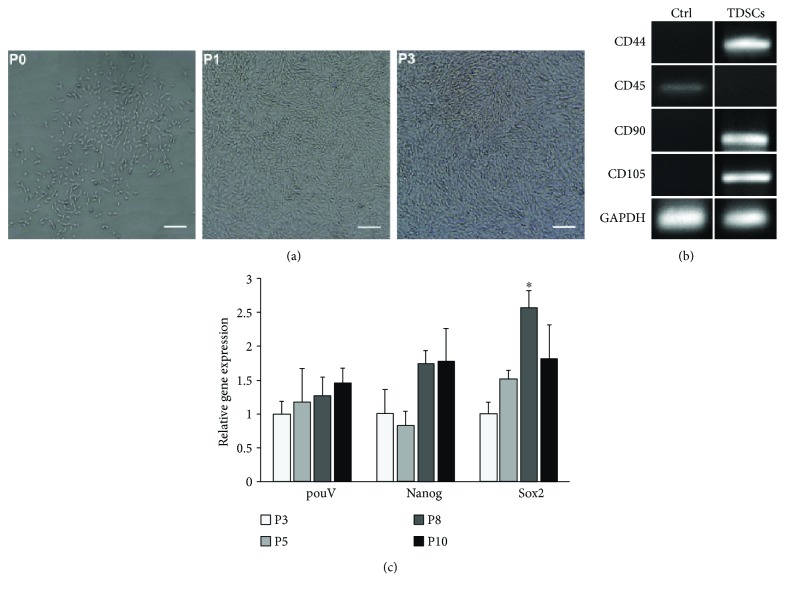
(a) Photomicrographs show different cell morphologies at different passages. At P0, spindle-shaped and polygonal cells were observed, and P1 cells demonstrated spindle-shaped fibroblastic morphology. At P3, homogeneous fibroblast-like cells were observed. Scale bars: 200 *μ*m. (b) Phenotype of tendon-derived cells. RT-PCR was performed using total RNA extracted from P3 cells. The right column shows the results with total RNA from TDSCs. Total RNA extracted from turkey white blood cells was used as a control (left column). (c) Gene expression analysis of embryonic stem cell markers *PouV*, *Nanog*, and *Sox2* at different cell passages. ^∗^*P* < 0.05 as compared to P3 cells.

Spindle-shaped and polygonal cells were both found at P0. At P1, cells demonstrated spindle-shaped fibroblastic morphology. The majority of cells at P3 retained fibroblast-like morphology Figure 2(a)).

### 4.3. Phenotype of Tendon-Derived Cells

The expression of MSC phenotypic markers was evaluated using RT-PCR (Figure 2(b)). Our results showed that the tendon-derived cells expressed surface antigens CD44, CD90, and CD105, but not CD45, thus indicating the mesenchymal lineage origin of these cells.

### 4.4. Expression of PouV, Nanog, and Sox2 Transcription Factors

The gene expression of *PouV*, *Nanog*, and *Sox2* was detected up to passage 10. P8 cells expressed higher levels of *Sox2* (*P* = 0.011) than did P3 cells. No significant difference was found in the mRNA expression of *PouV* (*P* = 0.792) or *Nanog* (*P* = 0.136) between different passages (Figure 2(c)).

### 4.5. Expression of Tenogenic Markers

**Figure 3:**
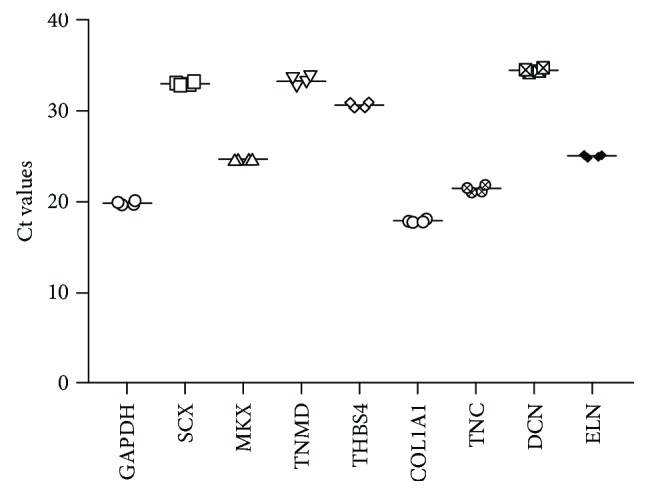
Scatter plot showing the mRNA expression of eight tendon-related genes in turkey TDSCs. The horizontal bar represents the mean cycle threshold (Ct) value of each gene. *GAPDH* serves as endogenous control. *SCX*: scleraxis; *MKX*: mohawk; *TNMD*: tenomodulin; *THBS4*: thrombospondin-4; *TNC*: tenascin C; *COL1A1*: collagen type I; *DCN*: decorin; *ELN*: elastin.

The gene expression level of tendon-related markers was examined by qRT-PCR. The isolated turkey TDSCs had high mRNA expression level of *MKX*, *COL1A1*, *TNC*, and *ELN* (Figure 3).

### 4.6. Osteogenic Differentiation Potential

**Figure 4:**
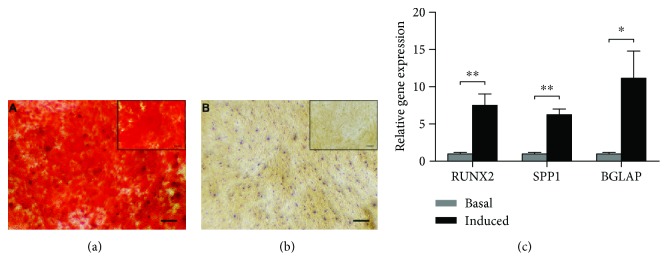
Osteogenic induction evaluated with Alizarin red S staining after 21 days in osteogenic media (a) or basal (b) media. Calcium nodules were seen in osteogenic medium (a), but not in basal medium (B). Scale bars: 200 *μ*m; inset, 100 *μ*m. (c) Graph showing the osteogenic gene (*RUNX2*, *SPP1*, and *BGLAP*) expression compared between osteogenic medium and its respective basal cultures. The level of expression of each target gene was normalized to *GAPDH*. ^∗∗^*P* < 0.01 and ^∗^*P* < 0.05. *RUNX2*: runt-related transcription factor 2; *SPP1*: osteopontin; *BGLAP*: osteocalcin.

After 21 days of osteogenic induction, Alizarin Red S assay demonstrated that induction cultures had calcium nodules (Figure 4(a)), which were absent in the basal cultures (Figure 4(b)). The mRNA expression level of *RUNX2* (*P* = 0.0028), *SPP1* (*P* ≤ 0.001), and *BGLAP* (*P* = 0.011) was also upregulated after 21 days of incubation in osteogenic medium (Figure 4(c)).

### 4.7. Adipogenic Differentiation Potential

**Figure 5:**
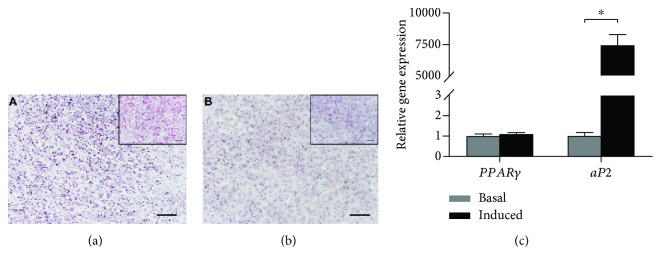
Adipogenic potential was determined by Oil red-O staining with hematoxylin counterstaining after culturing for 21 days in adipogenic media (a) or basal (b) media. Cytoplasmic lipid droplets were seen in adipogenic medium (a), but not in basal medium (b). Scale bars: 100 *μ*m; inset, 50 *μ*m. (c) Adipogenic gene (*PPARγ* and *aP2*) expression compared between osteogenic medium and its respective basal cultures. The level of expression of each target gene was normalized to *GAPDH*. ^∗^*P* < 0.01. *aP2*: adipocyte-binding protein 2; *PPARγ*: peroxisome proliferator-activated receptor.

Oil red-O staining showed lipid droplet accumulation within the cells upon adipogenic induction for 3 weeks (Figure 5(a)). This was absent in the control group (Figure 5(b)). The gene expression level of *aP2* (*P* ≤ 0.001) was significantly upregulated, whereas there was a trend of increased expression of *PPARγ* (*P* = 0.075) after adipogenic differentiation for 21 days (Figure 5(c)).

### 4.8. Chondrogenic Differentiation Potential

**Figure 6:**
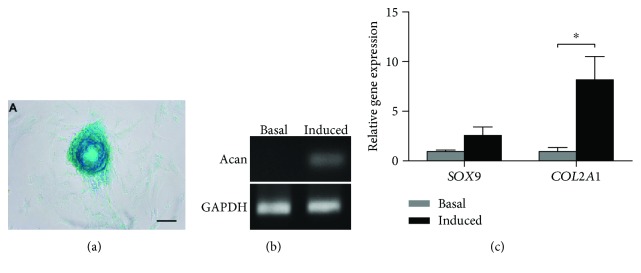
(a) Chondrogenic potential was evaluated by Alcian blue staining of proteoglycan in micromass pellets after culturing in chondrogenic medium for 21 days. Scale bars: 50 *μ*m. (b) Ethidium bromine gel of *ACAN* showed no expression of *ACAN* products for basal cultures. (c) Chondrogenic gene (*SOX9* and *COL2A1*) expression compared between chondrogenic medium and its respective basal cultures. The level of expression of each target gene was normalized to *GAPDH*. ^∗^*P* < 0.05. *SOX9*: sex-determining region Y-box9; *COL2A1*: collagen type II; *ACAN*: Aggrecan.

After 21 days of chondrogenic induction, there was glycosaminoglycan deposition found in micromass by Alcian blue staining (Figure 6(a)). There was increased gene expression of *COL2A1* (*P* = 0.03), whereas expression of *SOX9* showed a trend (*P* = 0.071) to increase after chondrogenic induction for 3 weeks (Figure 6(c)). The ethidium bromine gel showed a thick band for induction culture and no *ACAN* expression for basal culture (Figure 6(b)).

## 5. Discussion

This study demonstrated that turkey flexor tendon harbors a population of cells that has stem cell characteristics. Using methods previously described [23, 24, 35], we have isolated for the first time multipotent cells from the turkey flexor tendon. The cells were plastic adherent, possessed high proliferative potential, and were able to form colonies and have multilineage potential. When plating at 500 cells/cm^2^, about 4% of nucleated cells formed adherent cell colonies. These findings corresponded well with previous studies that have shown plating cells at low density allowing the selective expansion of stem cells from tendon [23, 35]. Furthermore, the percentage of stem cells was comparable to that shown in previous studies [26, 35]. Bi et al. [26] showed that about 3% to 4% of cells derived from mouse patellar tendons and human hamstring tendons were TDSCs. Rui et al. [35] showed that rat flexor tendons contained approximately 1% to 2% of TDSCs. In contrast, the percentage of MSCs residing in the adult bone marrow is less than 0.01% [15, 37, 39]. Therefore, tendon could be an alternative tissue for providing sources of MSCs.

Our group has recently compared the turkey flexor tendon to commonly used animal models and human hands [40]. It was found that turkey flexor tendon has more similarities to human than canine and chicken in terms of structure, function, and mechanical properties. Previous assumptions were made that since turkey leg tendons mineralize, their flexor tendons would mineralize likewise and thus would not make an ideal flexor tendon model [41–43]. However, we have determined that the tendon in the digit is not calcified, which is essential for flexor tendon research. Given that the 3rd digit of the turkey has the most suitable scale for experimentation [33] and zone II flexor tendon injuries are difficult to repair [6, 44], we focused our evaluation of the biological potential of these tendons.

The TDSCs isolated from turkey tendon were positive for MSC markers CD44, CD90, and CD105, while lacking expression of CD45 (a marker of all hematopoietic cells) [45]. These phenotypic profiles fulfilled the requirements proposed by the International Society for Cellular Therapy (ISCT) for defining MSCs [46]. Similarly, surface antigens CD44 and CD90 were also seen in mouse and human TDSCs [26] and in rat TDSCs [35]. Currently, there are no known turkey protein antibodies for CD44, CD90, CD105, and CD45 available; we attempted to use rat anti-mouse monoclonal antibodies to examine the phenotype of cells by flow cytometry, but we did not observe species cross-reactions. In addition, turkey TDSCs exhibited high mRNA expression of tenogenic markers, including *MKX*, *COL1A1*, *TNC*, and *ELN*. *MKX* is essential for tendon differentiation and collagen fibril maturation [47]. *COL1A1*, *TNC*, and *ELN* are regarded as major markers of the tendon extracellular matrix [48]. The gene expression of embryonic stem cell markers, including *PouV*, *Nanog*, and *Sox2* in TDSCs, were also evaluated. *PouV* is a homologue of *Oct4* in mammals and plays a key role in regulating chicken embryonic stem cell stemness [49]. *Nanog* and *Sox2* are also involved in the maintenance of stemness in undifferentiated embryonic stem cells [50, 51]. Our results showed that the expression of *PouV*, *Nanog*, and *Sox2* were detected in turkey TDSCs up to 10 passages *in vitro* without significantly reduced expression. This was comparable with previous studies that gene expressions of *PouV*, *Nanog*, and *Sox2* can be detected up to passage 8 in chicken BMSCs [36]. This is further indication that TDSCs maintain stem characteristics. The increased *Sox2* expression in P8 cells could be postulated as a result of the enrichment of stem cells with *in vitro* expansion while preserving their stemness. It has been suggested that current methods are incapable of isolating pure TDSCs at the early passage stage [52]. With the passage increased, the population of stem cells increased relative to the non-stem-cell population. Moreover, TDSCs from rat patellar tendons demonstrated higher clonogenicity with *in vitro* passaging [53], indicating that cell subculture might lead to increased expression of *Sox2* which is critical for MSC self-renewal capacity [54].

To characterize the multipotency of the turkey TDSCs, we differentiated the tendon-derived cells for osteogenesis, chondrogenesis, and adipogenesis and found that tendon-derived cells had multilineage capacity. Further, we evaluated the gene expression of lineage-specific markers for trilineage differentiation. Differentiation of TDSCs into osteogenic lineage was confirmed by matrix mineralization and significant upregulation of bone markers *SPP1*, *RUNX2*, and *BGLAP*. Furthermore, adipogenic differentiation was shown by Oil red-O staining of lipid droplets within cells and by increases in *aP2* gene expression, which is a late marker for adipocyte [55] but not *PPARγ*, which induced early during adipocyte differentiation [56]. The chondrogenic potential of TDSCs was demonstrated by synthesis of proteoglycans using Alcian blue stain analysis. The expression of *COL2A1* and *ACAN* (major cartilage extracellular matrix components) [57] was upregulated but not *SOX9*, an early marker for chondrogenesis [58] in micromass cultures. Our findings regarding expression of *PPARγ* and *SOX9* were different from previous studies by Rui et al. [35] who found an upregulation of *PPARγ* and *SOX9* in rat TDSCs after adipogenic and chondrogenic induction, respectively. However, the negative feedback mechanisms as well as different species might account for the differences.

In conclusion, we have for the first time isolated and shown that TDSCs from turkey exhibit clonogenicity, MSC marker expression, and multilineage differentiation potential. The successful isolation of tendon-derived stem cells from turkey should prove to be an important model system for future research in tendon tissue engineering in terms of structure, function, and biology.

## Figures and Tables

**Table 1:** Sequences of primers used for reverse transcription polymerase chain reaction.

Gene	Primer	5′-sequence-3′	Product size (bp)	Accession no.
GAPDH	Fwd Rev	TGGGAAGCTTACTGGAATGG CTTGGCTGGTTTCTCCAGAC	88	NM_204305.1
CD44	Fwd Rev	GGTTTTATAGTGGGGCATATTGTTATCCC TTAACCGCGATGCACACGGC	700	AF153205
CD45	Fwd Rev	CACTGGGAATCGAGAGGAAA CTGGTCTGGATGGCACTTTT	574	NM 204417
CD90	Fwd Rev	GGTCTACATGTGCGAGCTGA AAAGCTAAGGGGTGGGAGAA	471	NM 204381
CD105	Fwd Rev	ACGGATGACACCATGGAAAT ATGAGGAAGGCTCCAAAGGT	704	AY702002
PPAR*γ*	Fwd Rev	GGATTCATGACACGGGAGTT GCGTTGAACTTCACAGCAAA	92	NM_001001460.1
aP2	Fwd Rev	GAGTTTGATGAGACCACAGCAGA ATAACAGTCTCTTTGCCATCCCA	312	AF432507
RUNX2	Fwd Rev	CAGGCATGTCACTGGGTATG TATGGAGTGCTGCTGGTCTG	115	NM_204128.1
SPP1	Fwd Rev	AGCCACCACACACACAGGTA TGAAGCCAGGTCATTCTGTG	87	M59182.1
BGLAP	Fwd Rev	CGCAGTGCTAAAGCCTTCAT CTCAGCTCACACACCTCTCG	140	NM_205387.1
SOX9	Fwd Rev	CTCAAGGGCTACGACTGGAC GTACTGGTCAGCCAGCTTCC	141	NM_204281.1
COL2A1	Fwd Rev	AAGGGTGATCGTGGTGAGAC TCGCCTCTGTCTCCTTGTTT	107	AY046949.1
ACAN	Fwd Rev	ACTCCCGACACAACATCACA TGCGCTAGTTCAACATCTGG	101	NM_204955.2
PouV	Fwd Rev	TACATGCCACCTTTCCACAA CAGTGGCTGCTGTTGTTCAT	80	NM_001309372.1
Nanog	Fwd Rev	TTGGAAAAGGTGGAACAAGC GGTGCTCTGGAAGCTGTAGG	140	NM_001146142.1
Sox2	Fwd Rev	GCCCTGCAGTACAACTCCAT CCTTGCTGGGAGTACGACAT	83	NM_205188.2
SCX	Fwd Rev	TCCAGCTACATCTCCCACCT GCTGGGAGTTCTCGGAGTC	145	NM_204253.1
MKX	Fwd Rev	GTTGGGCTTTGCGAATAAAA ACGAGTCATCACTGCTCACG	81	XM_019616306.1
TNMD	Fwd Rev	CGGCGAGAAGAAGAAAATTG CTCCAGGATCTCCTCAGTGC	91	XM_003208349.3
THBS4	Fwd Rev	ATGCTCAGATTGACCCCAAC CCCTCGAAGTCAACACCATT	121	XM_019610190.1
COL1A1	Fwd Rev	CTGAAGAAGGCTCTGCTGCT CATGCTCCAGTGTGACTCGT	116	XM_015273228.1
TNC	Fwd Rev	GCCCATGGAGTTCAACATCT TGTAGCCGCAGCACTTATTG	136	NM_205456.4
DCN	Fwd Rev	CAACACCAAAAAGGCAACCT CTGCAGAGCGTTCATGGATA	107	NM_001030747.2
ELN	Fwd Rev	TGGCTATAGATTGCCCTTCG CCAACACCTGTCCCAGTAGG	99	NM_001293107.1
